# Conditional Survival in Prostate Cancer in the Nordic Countries Elucidates the Timing of Improvements

**DOI:** 10.3390/cancers15164132

**Published:** 2023-08-16

**Authors:** Frantisek Zitricky, Asta Försti, Akseli Hemminki, Otto Hemminki, Kari Hemminki

**Affiliations:** 1 Biomedical Center, Faculty of Medicine, Charles University Pilsen, 30605 Pilsen, Czech Republic; 2 Hopp Children’s Cancer Center (KiTZ), 69120 Heidelberg, Germany; 3 Division of Pediatric Neurooncology, German Cancer Research Center (DKFZ), German Cancer Consortium (DKTK), 69120 Heidelberg, Germany; 4 Cancer Gene Therapy Group, Translational Immunology Research Program, University of Helsinki, 00290 Helsinki, Finlandotto.hemminki@helsinki.fi (O.H.); 5 Comprehensive Cancer Center, Helsinki University Hospital, 00029 Helsinki, Finland; 6 Department of Urology, Helsinki University Hospital, 00029 Helsinki, Finland; 7 Division of Cancer Epidemiology, German Cancer Research Center (DKFZ), Im Neuenheimer Feld 580, 69120 Heidelberg, Germany

**Keywords:** prognosis, periodic survival, PSA screening, treatment

## Abstract

**Simple Summary:**

Prostate cancer (PC) is the most common male cancer, and the numbers of new cases increased hugely when prostate-specific antigen (PSA) testing became commonplace. The consequence was that the diagnostic age shifted toward younger men with less-advanced PC. Such changes are known to improve cancer survival, and in the Nordic countries, the 5-year survival for PC increased from about 60% to 90%; however, since testing stabilized, this improvement has slowed, and the 5-year survival had reached 95% by the year 2020. By analyzing survival in different periods after diagnosis, we observed that the most critical time for death was between years 1 and 5, assumably because of metastatic deaths. Some metastases are difficult to detect at diagnosis, and some arise later in the course of the disease. For continued survival, improvements in early diagnosis and more effective treatment will be required.

**Abstract:**

Background: The incidence of prostate cancer (PC) increased vastly as a result of prostate-specific antigen (PSA) testing. Survival in PC improved in the PSA-testing era, but changes in clinical presentation have hampered the interpretation of the underlying causes. Design: We analyzed survival trends in PC using data from the NORDCAN database for Denmark (DK), Finland (FI), Norway (NO) and Sweden (SE) by analyzing 1-, 5- and 10-year relative survival and conditional relative survival over the course of 50 years (1971–2020). Results: In the pre-PSA era, survival improved in FI and SE and improved marginally in NO but not in DK. PSA testing began toward the end of the 1980s; 5-year survival increased by approximately 30%, and 10-year survival improved even more. Conditional survival from years 6 to 10 (5 years) was better than conditional survival from years 2 to 5 (4 years), but by 2010, this difference disappeared in countries other than DK. Survival in the first year after diagnosis approached 100%; by year 5, it was 95%; and by year 10, it was 90% in the best countries, NO and SE. Conclusions: In spite of advances in diagnostics and treatment, further attention is required to improve PC survival.

## 1. Introduction

The incidence of prostate cancer (PC) increased vastly upon the introduction of prostate-specific antigen (PSA) testing in the public domain, with concomitant changes in the clinical presentation of PC. In the Nordic countries, opportunistic PSA testing began in the late 1980s/1990, but it began later in Denmark [1,2]. Clinical changes in the PSA era included a lower diagnostic age, a lower T stage and a lower proportion of patients presenting with distant metastases [2,3]. The vast increase in the incidence of PC with stable or decreasing mortality raised concerns about overdiagnosis, which has been estimated to vary from 10 to 80% depending on many factors, such as age at testing and PSA level [2,4,5].

In Sweden, nation-wide data on the principle reasons for a diagnosis of PC are available from 2004 onwards (National quality register for prostate cancer, https://statistik.incanet.se/npcr/ (accessed on 22 June 2023)); 28.6% of patients were non-symptomatic men diagnosed due to elevated PSA levels, and this proportion increased to 52.9% in 2020. Lower urinary tract symptoms and other symptoms accounted for more than 30% each in 2004, and by 2020, both accounted for no more than 20% of the diagnosed cases of PC. The PSA level may also increase in cases of benign prostatic hyperplasia, which is one of the most common urological diseases affecting elderly men and often requires surgical treatment [6]. Unfortunately, PSA determination cannot distinguish between cancer and hyperplasia, which is one of the reasons for the overdiagnosis of PC. Nevertheless, the diagnostic PSA level is used in risk stratification and treatment planning for PC patients (https://statistik.incanet.se/npcr/ (accessed on 22 June 2023)) [7,8]. 

In the Nordic countries, between 7 and 14% of PC patients have been diagnosed with metastases (de novo/synchronous, M1 in TNM staging), but for a large proportion of patients, the metastatic status remains unverified at diagnosis (Mx) [9,10]. Cancer registries consider metastases only at the time of diagnosis, and information on metastases (recurrent and metachronous) that appear later is limited; this is also true of many clinical studies which do not specify the timing of recurrent metastases. A US estimation of PC metastases assigned 45% to de novo and 55% to recurrent types, and the same proportions were found in a patient cohort [11,12]. A Swedish study covering the years 1987–2006 found that in 50% of PC deaths, the cause was assigned to PC [13]. The longer PC patients survive after diagnosis, the larger the proportion of deaths are assigned to non-cancer causes [14]. Data from the Swedish hospital discharge register showed that 89% of all PC metastases (including multiple metastases in the same patient) were located in the bone, 10% in the liver and 7% in the lung [15]. According to that study, which investigated the bone metastases of all common cancers, about 75% of bone metastases originated from PC among male cancers diagnosed at an age of more than 70 years. Bone scanning has been the common means of diagnosing metastatic PC.

A Danish study analyzed the clinical characteristics of patients who died of PC in two periods: 1995–1999 and 2009–2013 [16]. The proportion of metastatic tumors decreased from 49.4% to 38.3%, while the proportion of locally advanced tumors (clinical T3-4 and/or N+ and M0) increased from 8.6% to 27.3%; the median survival increased from 1.11 to 2.15 years in the metastatic group and from 1.41 to 3.75 years in the locally advanced group. As in this study, the increasing survival of metastatic patients has been reported in other Nordic studies. The median survival from 2010 to 2015 was 2.7 years in Sweden, and from 2015 to 2018 it was 3.3 years in Norway [9,10]. The traditional treatment is androgen deprivation therapy (ADT), and for castration-resistant tumors, several new drugs have become available [8,9,10]. Some 10% of PC patients have been diagnosed with locally advanced tumors, characterized by T3 or T4 (PSA < 100 ng/mL); among the patients diagnosed in the 2008–2011 period, 83% survived for 5 years [17]. In Sweden, some 15% of patients with locally advanced tumors received radical treatment in the year 2000, but this increased in 15 years to over 40%. Radical radiotherapy (with ADT) was more commonly applied than radical prostatectomy, for which robotic surgery was introduced after the year 2000 [8,17]. 

Survival is commonly reported for up to 1 or 5 years and sometimes up to 10 years. The routine 1- and 5-year survival data were sufficient at times when most cancer patients died within 5 years after diagnosis [18]. The situation has completely changed in the past 50 years, and in the Nordic countries, the relative 5-year survival exceeds 60% for most solid cancers [18]. With increasing survival times, we must be aware of the life-threatening periods for patients beyond years 1 and 5. Conditional survival is a useful survival metric for this purpose as it estimates survival probabilities in those who have already survived X years [19]. In fatal cancers, deaths are often due to metastases, but in cancers such as PC, for which many metastases appear after diagnosis, conditional survival may pinpoint critical periods. Conditional survival has become increasingly important in clinical survival estimation through its relationship to event-free survival [20,21]. In the present study, we assessed relative PC survival rates in Denmark (DK), Finland (FI), Norway (NO) and Sweden (SE) over a period of 50 years, until 2020. Cancer registration was initiated early in these countries and is generally characterized by high coverage and minimal loss to follow-up [22]. We obtained PC survival data from the NORDCAN database for 1-, 5- and 10-year relative survival and developed conditional survival data for the years 2 to 5 (5/1), 5 to 10 (10/5) and 2 to 10 (10/1), allowing for the assessment of changes in survival at various intervals in the four countries and correlations with known developments in PC diagnostics and treatment.

## 2. Methods

The source of the data on the incidence and survival of PC was the NORDCAN database 2.0, and we examined data from the years 1971 to 2020; the database was accessed in the winter of 2023 [22,23]. The database is located at the International Agency for Cancer (IARC) and was accessed at the following website: https://nordcan.iarc.fr/en (accessed on 22 June 2023) [24]. Relative survival data for 1-, 5- and 10-year survival were obtained. The NORDCAN 5- and 10-year survival data are based on the cohort survival method for all but the last period, for which the hybrid method is applied [25,26]. Age standardization for relative survival applies the Pohar Perme estimator, using national life tables to derive the expected rates [27]. Age groups 0 to 89 were considered. 

For statistical modeling and data visualizations, R statistical software (https://www.r-project.org (accessed on 22 June 2023)) was used in the R studio environment (https://posit.co/ (accessed on 22 June 2023)) [28]. Relative survival trends (NORDCAN 5-year periodic %) were generated using Gaussian generalized additive models (GAMs) with thin plate regression splines in a Bayesian framework [28]. The methods for the estimation of the conditional relative survival are described elsewhere [28]. Changes in survival trends were estimated through annual % changes and through “breakpoints”, which marked times at which the annual changes in survival could be defined with at least 95% plausibility. These are described in the legends for the figures, and the detailed estimation methods are available in Reference [28]. 

The approximate initiation of opportunistic PSA testing in FI, NO and SE was around 1990, despite the national authorities’ recommendations against screening [29]. Such a recommendation probably caused the delay in the initiation of PSA testing in DK until about 1995 [29]. 

Other recent survival data were available up to the year 2018 for White men from the USA, including Hispanics, through the US Surveillance, Epidemiology and End Results (SEER), which was accessed in the winter of 2023 at the following website: https://seer.cancer.gov/statistics-network/explorer/application.html?site=1&data_type=1&graph_type=2&compareBy=sex&chk_sex_3=3&chk_sex_2=2&rate_type=2&race=1&age_range=1&hdn_stage=101&advopt_precision=1&advopt_show_ci=on&hdn_view=0&advopt_display=2#graphArea (accessed on 22 June 2023).

## 3. Results

Numbers of PC patients are shown for 1971–75 and 2016–20 in the Nordic countries (Table 1). The number of cases increased the most for FI, increasing by 7.6-fold, and the least for SE, demonstrating a 3.1-fold increase between the two periods.

**Figure 1:**
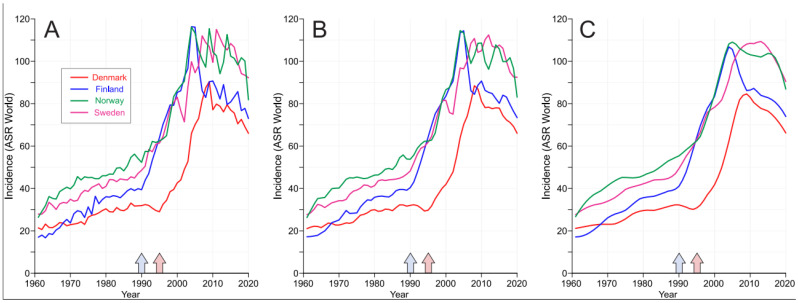
Age-standardized incidence in prostate cancer in the Nordic countries, showing raw incidence data (**A**) and smoothened data with bandwidths of 0.1 (**B**) and 0.2 (**C**). The approximate starting times for opportunistic PSA screening are shown by arrows on top of the x-axes (the first arrow is for FI, NO and SE, and the second one is for DK).

The age-standardized (world) incidence of PC for each Nordic country is shown in Figure 1. The plots show the raw incidence data (A) and the smoothened data with bandwidths of 0.1 (B) and 0.2 (C). The approximate initiation times for opportunistic PSA screening are shown by arrows on top of the x-axes. For FI and NO, sharp incidence peaks emerged in 2003; in SE, the first discrete peak occurred in 2007, and in DK, a sharp peak emerged in 2008. In the raw data, the discrete peaks may indicate random variations or regional introductions of PSA screening [30]. 

**Figure 2:**
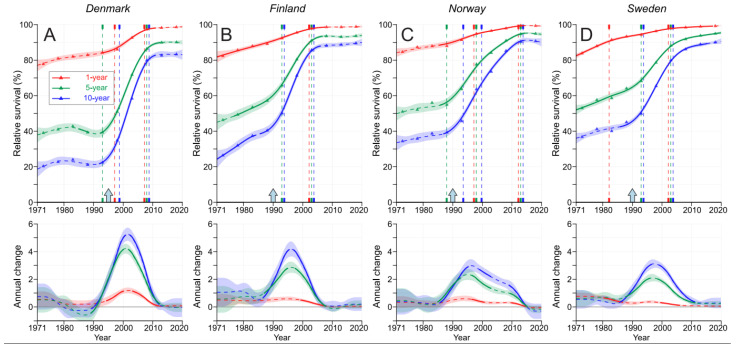
Relative 1-, 5- and 10-year survival in DK (**A**), FI (**B**), NO (**C**) and SE (**D**). The vertical lines mark significant changes in the survival trends (“breakpoints”), and the bottom curves show the estimated annual changes in survival. The curves are solid if there is >95% plausibility of the growth or decline. Shadow areas indicate 95% credible intervals. All curves are color-coded (see the insert). The approximate starting times for opportunistic PSA screening are shown by arrows on top of the *x*-axes.

Relative 1-, 5- and 10-year surviva for PC are shown in Figure 2 for each Nordic country; the exact values are shown in Supplementary Table S1. For FI, NO and SE, the 1-year survival started at over 80% (in DK, it was below 80%) and had approached 100% by the year 2010. In NO and SE, the 5- and 10-year survival curves were quite similar, with upward shifts occurring around the introduction of PSA screening in 1990, after which the average annual improvements reached 2% for 5-year survival and 3% for 10-year survival. These improvements stagnated by 2010. In FI, and particularly in DK, the shapes of the curves resembled those for NO and SE, but as the starting levels were lower, the annual increases were steeper; in DK, they were 4% for 5-year survival and over 5% for 10-year survival. In DK, the 5- and 10-year survival curves remained stable until 1990. In FI, the curves had already plateaued after the year 2000, and in DK, the final plateaus remained below those of the other countries. 

**Figure 3:**
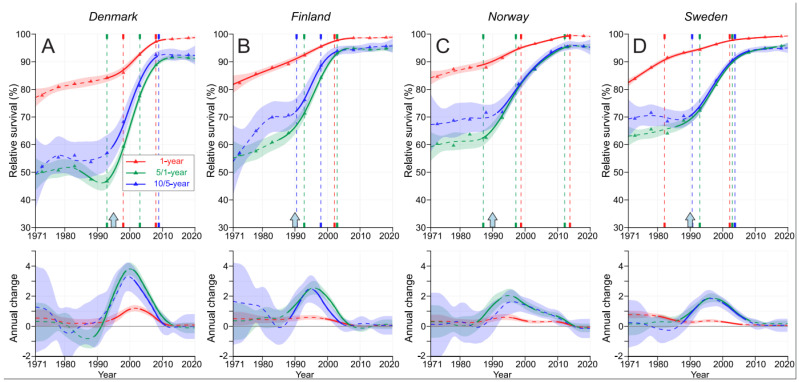
Relative 1-, 5/1- and 10/5-year survival in DK (**A**), FI (**B**), NO (**C**) and SE (**D**). The vertical lines mark significant changes in the survival trends (“breakpoints”), and the bottom curves show the estimated annual changes in survival. The curves are solid if there is >95% plausibility of the growth or decline. Shadow areas indicate 95% credible intervals. All curves are color-coded (see the insert). The approximate starting times for opportunistic PSA screening are shown by arrows on top of the x-axes.

In Figure 3, we plot the 1-year relative survival together with the conditional 5/1-and 10/5-year relative survival to allow for a stepwise assessment of survival in year 1, between years 2 and 5 and further, between years 6 and 10; the exact values are shown in Supplementary Table S2. The curves for conditional survival did not improve until 1990 except in FI. At all times, the conditional 10/5-year survival was on top of the 5/1-year survival, with the largest margin in DK, a smaller margin in FI and a diminishing margin in NO and SE after the year 2000. The annual changes were the largest in DK, but these peaks occurred about 5 years later than the peaks in the other countries. 

**Figure 4:**
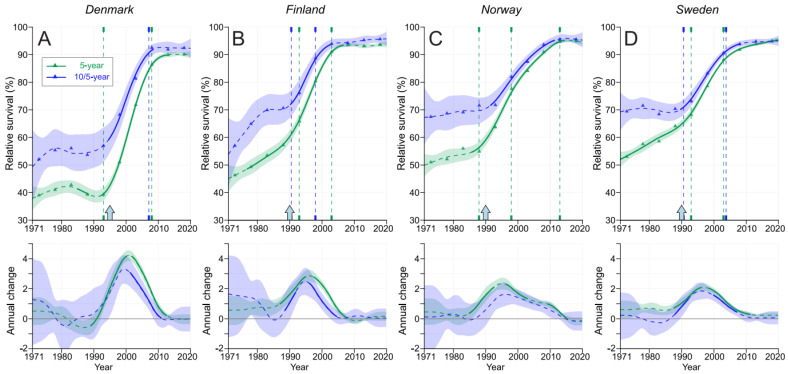
Relative 5- and 10/5-year survival in DK (**A**), FI (**B**), NO (**C**) and SE (**D**). The vertical lines mark significant changes in the survival trends (‘breakpoints”), and the bottom curves show the estimated annual changes in survival. The curves are solid if there is >95% plausibility of the growth or decline. Shadow areas indicate 95% credible intervals. All curves are color-coded (see the insert). The approximate starting times for opportunistic PSA screening are shown by arrows on top of the x-axess.

In Figure 4, we plot the 5-year survival together with the conditional 10/5-year survival. The starting levels in DK and FI were below those of NO and SE but with steeper increases, and the final plateau was approximately equal for countries other than DK, which shows a lower level. The curve for 10/5-year survival was on top of 5-year curve, with the largest margins in DK and FI. 

Supplementary Table S1 shows the exact survival data for 1-, 5- and 10-year survival in these countries. SE showed the highest 1-year survival for most periods, but it was overtaken by NO for the last two periods, with the best final survival of 99.3%. However, the final results for all countries were within 0.7 % units. SE showed the best 5-year survival in the initial period, but the best final survival of 94.9% was a tie between SE and NO. The final 5-year survival for DK was only 90.1%. Regarding 10-year survival, SE advanced well until 1990 and was then taken over by FI until 2010 and in the final periods by NO, finishing at 90.7%, a value well over DK’s survival at 83.3%. An indication of the plateau in the increases in 5- and 10-year survival toward the end can be seen as very few significant increases toward the last 10-year period. 

Supplementary Table S2 lists the conditional survival valies for all countries. The best conditional survival periods were shared by NO (few) and SE and FI (most), with little differences between these countries in the end, which were significantly better than the conditional survival of DK. A conspicuous detail is the stagnation of conditional survival, with few improvements made in the recent 15 year period.

Comparisons with the US SEER data for the years 2014–18 for White men (including Hispanics) showed a 1-year relative survival of 99.1% and a 5-year survival of 97.1%. 

## 4. Discussion

PC survival in the Nordic countries showed a modest increase or no increase in the pre-PSA-screening era, a large transition upwards within 15 years of the introduction of PSA screening and another plateau or modest increase from 2010 onwards (Figure 2). In the pre-PSA-screening era, survival differed extensively between the 1-, 5- and 10-year metrics. During the implementation of PSA screening, the differences between these three survival metrics narrowed, and in the final period, the 1-year survival approached 100%, and the difference between the 5- and 10-year survival rates had stabilized to about 5 % units. For the pre-PSA-screening era, the present results show that conditional 10/5-year survival from the years 6 to 10 (5 years) was about 15 % units better than survival in the first 5 years (Figure 4). During the implementation of PSA screening, the difference between these survival metrics narrowed, and in the last 15 years, they merged in SE and narrowed to less than 2 % units in the other countries. In a global PC survival study covering the years 2010–2014, the Nordic countries FI, NO and SE were placed in the >90% category for 5-year survival [31]. The current results show further improvements for NO and SE, which are approaching 95%, while FI is at 94% and DK has surpassed 90%. However, even the best figures were below the present US 5-year survival of 97.1%; however, this rate was probably achieved via intense PSA testing [32]. 

The present novel observations for the pre-PSA-screening era until 1990 were the slow improvements in the 5- and 10-year survival rates in FI and SE, the marginal improvement in NO and the lack of improvement in DK. An additional novelty was the demonstration of the catching-up of the 5-year survival with the 10/5-year survival rates during the PSA-screening-implementation phase and the final culmination of these survival metrics. The changes could probably be largely rationalized by a complete change in the previous pool of PC patients, with a huge number of PSA-diagnosed early-onset PC patients (approximately a fourfold increase in patient numbers). PSA-tested patients were characterized by a low T stage and a low proportion of patients presenting with distant metastases [2,3]. According to the present results, in the pre-PSA year of 1980, about 15% of PC patients died in FI and NO during year 1 after diagnosis (somewhat less in SE and more in DK), 50% of patients died by year 5 and 65% died by year 10. In the post-PSA era of 2016–20, 1% of patients died by year 1, 5% by year 5 and 10% by year 10 (with a higher death rate in DK). In the last period, conditional survival data show that 1% of patients died by year 1, an additional 4% died from the years 2 to 5, and an additional 5% died from the years 6 to 10. A Korean relative survival study on PC patients diagnosed until 2013 showed improved survival and decreased mortality after 4 years post diagnosis [33]. The NORDCAN data extend to the year 2020, which implies that the present data are as up to date as is achievable by any national cancer registry. These data show that in the last 15 years, survival improvement has slowed down, probably indicating that PSA screening has reached its peak, and further survival improvements depend on novel gains in diagnostics, treatment and patient care. 

Improvements in survival have been reported for metastatic and locally advanced PC in the Nordic countries and in the Netherlands [9,10,17,34]. Survival in metachronous metastatic PC has been reported to be better than in synchronous metastatic PC [35]. ADT has been the basis of treatment for metastatic PC for decades, with few improvements made until the last 20 years. Several months of survival benefit were first shown with docetaxel and continued by enzalutamide, abiraterone and radiotherapy with 223Ra [9,10]. In recent years (not affecting our presented results), more treatments with survival benefits have been introduced and used earlier in the hormone-sensitive time space, including upfront triplet treatments (the use of ADT, docetaxel and either abiraterone or darolutamide) [8,36]. In locally advanced PC in SE, the use of radical radiotherapy and prostatectomy increased from 15 to 43% of patients in 15 years [17]. 

Simultaneously, fewer active treatments have been used in low-risk cancers as active surveillance has gained popularity [8,36]. While this has benefitted most patients in the form of fewer treatment-related adverse events, some patients might have missed the possibility of achieving a cure [37]. Knowledge of biopsy-related complications might also have led to more PSA-only or PSA- and MRI-based follow-ups [38]. These changes in practice, as well as the aging PC population, might explain why minimal improvements have been seen in the recent survival data points. Another plausible explanation is that the overall survival benefits seen in the selected trial populations have not thus far affected the epidemiological landscape in spite of positive reports [9,10]. 

In the Nordic countries, national guidelines for the diagnosis and treatment of PC are regularly updated, and these are greatly inspired by the guidelines of the European Society for Medical Oncology (ESMO) and the European Association for Urology (EAU) [8,36]. The guidelines recommend risk/stage adaptive therapies and diagnostics. Active surveillance is preferred in less-aggressive PC, while more aggressive local cancers should typically be treated with prostatectomy or radiotherapy [8,36]. In more advanced states, ADT and chemotherapy should be applied with the addition of novel agents in castration-resistant cases [8,36]. 

The limitations of the present study are the lack of any diagnostic (PSA and clinical) and pathological (TNM) information about cancers at diagnosis and any treatment data. It is, however, not feasible to assume that comparable pathological data were available over 50 years as even the closely collaborating Nordic cancer registries have difficulties in comparing data on tumor characteristics (stage) over the last decades [22,39]. A further limitation is that NORDCAN does not allow for survival analysis by age, which is an important determinant of survival. 

## 5. Conclusions

We showed shifts in PC-related relative survival coincident with the introduction of PSA testing. Relative 5-year survival was around 50% in the pre-PSA-screening era, and it increased to almost 95% in the post-PSA-screening era. Using conditional survival, the critical period was shown to be from year 1 to year 5 after diagnosis. As the major improvement, survival in the year 1 to 5 period almost reached the level of survival from year 6 to year 10. For PC, mortality in year 1 is low, but later mortality requires attention and is likely related to an unverified metastatic status at diagnosis or recurrent metastases.

## Figures and Tables

**Table 1:** Numbers of prostate cancer patients in the Nordic countries in the pre- and post-PSA periods.

Country	1971–1975	2016–2020	Increase, Fold
Denmark	4869	22712	4.7
Finland	3411	25990	7.6
Norway	6061	25412	4.2
Sweden	16561	52130	3.1

## Data Availability

A publicly available database was used.

## Supplementary Materials

The following supporting information can be downloaded at: https://www.mdpi.com/article/10.3390/cancers15164132/s1, Table S1 shows 1-, 5- and 10-year relative survival rates (95% confidence interval) for prostate cancer in the Nordic countries from 1971 to 2020 based on the NORDCAN database. Table S2 shows 5/1-, 10/5- and 10/1-year conditional survival rates for prostate cancer in Nordic countries from 1971 to 2020.
