# Supplementary material for: Conditional Survival in Prostate Cancer in the Nordic Countries Elucidates the Timing of Improvements
Source: Cancers (Basel). 2023 Aug 16;15(16):4132. doi: 10.3390/cancers15164132 (PMC10453103; doi:10.3390/cancers15164132)
Supplement: Supplementary file 1 [file cancers-15-04132-s001.zip › cancers-2494084-supplementary.pdf]

Supplementary Table S1. 1-, 5- and 10-year relative survival [95% confidence interval] for prostate cancer in the Nordic countries from 1971 to 2020 based on the NORDCAN database. * Significant increase between marked and the next period.

1-year	Denmark	Finland	Norway	Sweden
**1971-1975**	77.8[75.3-80.4]	82.3[79.0-85.8]	84.6[82.5-86.8]	83.8[82.8-84.9]*
**1976-1980**	80.9[79.2-82.6]	85.6[83.8-87.5]	87.3[85.4-89.2]	87.9[86.6-89.3]*
**1981-1985**	82.0[79.7-84.2]	87.9[86.0-89.9]	87.9[85.6-90.3]	91.5[90.5-92.6]
**1986-1990**	82.9[80.0-86.0]	89.1[87.1-91.1]	87.9[85.8-90.0]	93.3[92.5-94.0]
**1991-1995**	84.2[82.4-86.0]	92.3[91.0-93.7]*	91.4[89.7-93.1]*	94.3[93.5-95.0]*
**1996-2000**	86.0[83.9-88.1]*	95.5[94.6-96.4]*	94.9[94.2-95.6]*	96.3[95.9-96.7]*
**2001-2005**	92.6[91.5-93.8]*	97.8[97.4-98.2]*	96.5[96.1-96.9]*	97.9[97.6-98.2]
**2006-2010**	97.3[96.9-97.7]*	98.6[98.3-98.9]	97.9[97.5-98.3]*	98.4[98.2-98.7]
**2011-2015**	98.2[97.9-98.5]	98.6[98.3-98.9]	99.4[99.1-99.6]	98.9[98.7-99.1]
**2016-2020**	98.6[98.3-98.9]	98.8[98.4-99.1]	99.3[99.1-99.6]	99.2[99.1-99.4]
**5-year**				
**1971-1975**	39.0[36.1-42.2]	46.3[42.9-50.0]	51.0[48.1-54.2]	53.0[51.1-55.0]*
**1976-1980**	41.0[38.1-44.2]	49.3[45.8-53.0]	52.1[48.7-55.8]	57.6[55.4-59.8]
**1981-1985**	42.8[39.9-45.8]	53.5[50.3-56.8]	56.0[52.7-59.4]	58.6[56.5-60.9]*
**1986-1990**	39.3[36.1-42.6]	57.2[54.0-60.6]*	54.9[52.0-57.9]*	64.1[62.2-66.0]*
**1991-1995**	39.3[36.6-42.2]*	65.8[63.2-68.5]*	63.7[61.4-66.1]*	68.2[66.5-70.0]*
**1996-2000**	51.0[48.5-53.6]*	80.4[78.7-82.1]*	77.6[76.1-79.1]*	78.6[77.5-79.8]*
**2001-2005**	71.8[70.0-73.7]*	91.1[90.2-92.0]*	84.1[83.0-85.2]*	88.2[87.5-89.0]*
**2006-2010**	86.4[85.4-87.4]*	93.5[92.7-94.3]	91.0[90.1-91.8]*	91.8[91.2-92.3]*
**2011-2015**	89.9[89.0-90.8]	93.1[92.3-93.9]	94.7[94.0-95.4]	93.8[93.3-94.3]*
**2016-2020**	90.1[89.3-91.0]	93.6[92.8-94.4]	94.9[94.2-95.6]	94.9[94.4-95.4]
**10-year**				
**1971-1975**	20.3[17.4-23.6]	26.4[23.0-30.3]	34.4[31.4-37.5]	36.8[34.7-39.1]
**1976-1980**	22.7[20.1-25.5]	32.0[28.8-35.6]	35.8[32.7-39.2]	41.2[39.0-43.5]
**1981-1985**	24.0[20.9-27.6]	37.4[34.2-40.9]	38.7[35.2-42.4]	40.1[38.0-42.3]*
**1986-1990**	21.1[18.3-24.4]	40.4[37.2-43.9]*	39.3[36.5-42.2]*	45.1[43.1-47.2]*
**1991-1995**	22.4[20.0-25.1]*	50.1[47.3-53.1]*	45.7[43.3-48.3]*	49.9[48.0-51.8]*
**1996-2000**	34.8[32.2-37.7]*	71.3[69.1-73.6]*	63.6[61.8-65.5]*	65.4[63.9-66.9]*
**2001-2005**	58.4[56.0-60.9]*	85.6[84.1-87.2]	73.5[71.9-75.2]*	79.9[78.9-81.0]*
**2006-2010**	79.9[78.2-81.7]	88.1[86.7-89.6]	85.2[83.8-86.6]*	86.1[85.2-87.1]*
**2011-2015**	82.6[80.7-84.5]	88.7[87.3-90.0]	90.4[88.9-91.9]	88.9[87.8-89.9]
**2016-2020**	83.3[81.5-85.1]	89.5[88.2-90.8]	90.7[89.2-92.2]	90.0[89.0-91.1]

Supplementary Table S2. 5/1-, 10/5- and 10/1-year conditional survival for prostate cancer in Nordic countries from 1971 to 2020.

5/1-year	Denmark	Finland	Norway	Sweden
**1971-1975**	50.1	56.3	60.3	63.2
**1976-1980**	50.7	57.6	59.7	65.5
**1981-1985**	52.2	60.9	63.7	64
**1986-1990**	47.4	64.2	62.5	68.7
**1991-1995**	46.7	71.3	69.7	72.3
**1996-2000**	59.3	84.2	81.8	81.6
**2001-2005**	77.5	93.1	87.2	90.1
**2006-2010**	88.8	94.8	93.0	93.3
**2011-2015**	91.5	94.4	95.3	94.8
**2016-2020**	91.4	94.7	95.6	95.7
**10/5-year**				
**1971-1975**	52.1	57.0	67.5	69.4
**1976-1980**	55.4	64.9	68.7	71.5
**1981-1985**	56.1	69.9	69.1	68.4
**1986-1990**	53.7	70.6	71.6	70.4
**1991-1995**	57.0	76.1	71.7	73.2
**1996-2000**	68.2	88.7	82.0	83.2
**2001-2005**	81.3	94.0	87.4	90.6
**2006-2010**	92.5	94.2	93.6	93.8
**2011-2015**	91.9	95.3	95.5	94.8
**2016-2020**	92.5	95.6	95.6	94.8
**10/1-year**				
**1971-1975**	26.1	32.1	40.7	43.9
**1976-1980**	28.1	37.4	41	46.9
**1981-1985**	29.3	42.5	44	43.8
**1986-1990**	25.5	45.3	44.7	48.3
**1991-1995**	26.6	54.3	50	52.9
**1996-2000**	40.5	74.7	67	67.9
**2001-2005**	63.1	87.5	76.2	81.6
**2006-2010**	82.1	89.4	87	87.5
**2011-2015**	84.1	90	90.9	89.9
**2016-2020**	84.5	90.6	91.3	90.7
